# Consistently longer silent gaps in autistic speaker pairs across three conversational contexts

**DOI:** 10.1038/s41598-026-53132-z

**Published:** 2026-05-15

**Authors:** Simon Wehrle, Malin Spaniol, Kai Vogeley, Martine Grice

**Affiliations:** 1 Institut für Linguistik—Phonetik, University of Cologne, Cologne, Germany; 2 Department of Psychiatry, University Hospital Cologne, Medical Faculty, University of Cologne, Cologne, Germany; 3 Cognitive Neuroscience, Institute of Neuroscience and Medicine (INM-3), Research Center Jülich, Jülich, Germany

**Keywords:** Turn-taking, Inter-autistic communication, Naturalistic interaction, Conversation, Silent gaps, Autism, Neuroscience, Psychology, Psychology

## Abstract

We investigated the timing of turn-taking in matched autistic compared to non-autistic pairs of adult speakers. Turn-taking—the organisation of who speaks when—is the fundamental mechanism of communicative interaction, and has been found to be remarkably fast and robust across different groups of speakers. We build on the only previous study of turn-timing in in matched autistic dyads, while also conducting one of the first within-speaker comparisons of turn-timing across different conversational contexts. We recorded 18 autistic and 28 non-autistic speakers in matched dyads, conversing in three sequential conversational contexts. We analysed 10,251 turn transitions using the measure of Floor Transfer Offset (comprising silent and overlapping transitions). Bayesian inferential modelling was used for statistical analysis. Our results show longer silent gaps 1) in autistic speaker pairs and 2) in the task-based conversational context. The finding of longer silent gaps in autism does not align with the only previous study on conversations between autistic adults. We discuss the importance of investigating naturalistic, face-to-face interaction and question the idea that there is a single, fixed target for conversational turn-timing. We argue that speaker pairs skilfully adjust their turn-taking rhythm to match both interpersonal and situational factors.

**Supplementary Information:**

The online version contains supplementary material available at 10.1038/s41598-026-53132-z.

## Introduction

The rapid exchange of speaker turns is a foundational element of conversational interaction, with interlocutors optimising the speed of exchanges to maximize efficiency, and doing so despite the great cognitive demands on processing and prediction that this entails for each speaker–hearer^1–6^. Rapid turn-timing, characterised by a preference for very short silent gaps between speakers—typically around 200 ms—appears to be a near-universal phenomenon, although subtle differences in turn-timing have been observed for e.g. non-native^7^ and autistic speakers^8,9^, as well as patients on the schizophrenia spectrum^10^, all of whom may face particular challenges in conversational interaction.

Interestingly, the influence of conversational context on turn-timing remains understudied, and, as far as we are aware, only one relevant systematic empirical analysis has been published^11^. Most quantitative analyses of the timing of turn-taking, including arguably the most influential work^1,2^, have generally been restricted to specific kinds of interactions like question–answer pairs or telephone calls. In-depth analysis of turn-timing in naturalistic, multi-modal, face-to-face interaction remains scarce.

The swift exchange of turns is highly demanding and requires the accurate prediction of an interlocutor’s next conversational move— a characteristic of particular relevance for autism (spectrum disorder; ASD). While most work on turn-taking in autism has reported longer silent gaps between speakers^9,12,13^ (but see recent results showing more overlaps in child–caregiver interactions^14^), the most relevant recent work, which is the only study on conversation in autistic–autistic dyads to date, showed no clear difference between groups, except in the earliest stages of the conversation^8^. Note, however, that the data in this study were limited to one fixed conversational context involving structured, task-based interaction. Additionally, the visual channel was blocked through an opaque screen.

The current study seeks to fill an important gap in the literature by building on the only study of turn-taking in conversations between autistic adults so far^8^ through an investigation of face-to-face interaction and the systematic comparison of different conversational contexts. We analysed one group of autistic and one group of non-autistic speaker pairs (dyads), each conversing in three different conversational contexts, from fully free and spontaneous to highly structured and task-based.

Dyads were recorded within diagnostic group (ASD–ASD; non-ASD–non-ASD) rather than in mixed dyads (ASD–non-ASD), for two primary reasons. First, studies on communication in autism using matched dyads are still limited, even though some of the relevant results have fundamentally challenged assumptions put forth in research limited to mixed dyads^8,15–18^. Second, only by focusing on disposition-matched dyads can we gain a deeper understanding of autistic communication without the potential confounding effects of differing cognitive styles in mixed dyads^18^.

While the evidence base is too limited to yield clear predictions, the key research questions of interest are: 1) do autistic dyads produce longer silent transitions between speakers than non-autistic speaker pairs, and 2) does conversational context influence turn-timing, within and across groups?

In short, the present study provides compelling evidence for an affirmative answer to both of the above questions: turn-timing was slower—with longer silent gaps—1) in the ASD group, across conversational contexts, and 2) in the task-based compared to free conversational contexts, across groups.

The next sections provide details on methods and results, followed by a discussion of implications for autistic-specific communication styles as well as universal patterns and the importance of conversational context in turn-taking.

## Methods

In this section, we provide details on the study participants, the experimental procedure, the data set under study, and the analysis tools used. The methods for the overarching project of which this study forms one part were pre-registered on OSF (https://osf.io/eum4n). The final study differs from the pre-registered plan in the following ways, all of which are due to difficulties with recruitment that could not be resolved within one year and after three rounds of active recruitment: we recruited fewer than the targeted 50 participants per group, we did not record same-sex dyads exclusively, and we have not (yet) recorded any mixed (autistic–non-autistic) dyads. Complete details and specifications of the setup and experimental procedure can be found in the OSF repository at https://osf.io/z5crx/.

### Participants

All participants in the ASD group had received a clinical diagnosis from the Autism Outpatient Clinic, Department of Psychiatry, University Hospital Cologne. Out of the 18 autistic participants, 16 had a diagnosis of Asperger syndrome (ICD-10: F84.5) and the remaining 2 a diagnosis of childhood autism (ICD-10: F84.0). Diagnoses were independently confirmed by two specialised clinicians and supplemented by comprehensive neuropsychological assessments. All non-ASD participants had no neurological or psychiatric disorders and were not undergoing any psychopharmacological treatment. All participants were fluent German speakers and had normal or corrected-to-normal hearing and vision.

Summary statistics by group on gender, age, verbal IQ (measured using *Wortschatztest WST*, which has been shown to correlate highly with both verbal and general intelligence^19,20^) and AQ (autism quotient^21^) of all 46 participants are presented in Table 1. There was a clear and robust effect for a higher AQ in the ASD group. All non-ASD participants scored below the suggested clinical threshold of 32^22^. Out of the 18 ASD participants, 4 also scored below this threshold—but note that this is not surprising taking into account previous work^22,23^. Crucially, all participants from our ASD group had received an autism diagnosis following extensive and established clinical tests. The effects of group (or lack thereof) for age, verbal IQ and AQ were confirmed with Bayesian modelling (for details see https://osf.io/z5crx/).

**Table 1:** Participant information by group.

Group	Gender (n)		Age (years)		Verbal IQ		AQ	
	Female	Male	Mean	SD	Mean	SD	Mean	SD
ASD	7	11	36	12	108	12	37	7
Non-ASD	13	15	38	13	106	10	15	5

### Materials and procedure

Data collection took place at the Institute for Linguistics, Department of Phonetics, University of Cologne, Germany, between May 2023 and May 2024. Upon arrival, participants were welcomed, received an overview of the study objectives and procedures, and provided written informed consent. Participants were then briefly introduced to their interlocutors (without disclosing diagnosis or any other personal details) and informed that they would engage in conversational interaction. They were then fitted with light-weight, naturalistic mobile eye-tracking glasses (*Pupil Invisible*) with attached high-quality microphones (AKG 417 PP). (Results on eye gaze will be discussed in forthcoming work; see Section "Discussion" .)

Participants received written and verbal instructions and then commenced with the interaction, consisting of three conversational contexts, each lasting 10 min, in a fixed order^24,25^: Introduction (getting to know each other), Tangram task (comparing geometric shapes to see whether they matched)^26–28^, and Discussion (of the Tangram task). These contexts were chosen to enable the systematic comparison of fully free conversation (Introduction), highly structured, task-based interaction (Tangram), and free conversation with a set topic and an established degree of common ground (Discussion). Communication that took place outside of these three specified contexts did not enter into analysis.

A wide range of questionnaires related to cognitive profiles, interaction style, rapport and personal traits was completed by participants following the completion of the conversational recordings (see full list at https://osf.io/z5crx/). Following questionnaire completion, participants were debriefed and compensated at a rate of 12 EUR/hour.

The study received approval from the local ethics committee of the Medical Faculty at the University Hospital Cologne, and adhered to the ethical standards set forth in the 1964 Declaration of Helsinki and its later amendments.

### Data and analysis

The following sections will provide details on the corpus, measurements and processing, and analysis. For the sake of brevity, some specific technical details are discussed with reference to open-access repositories containing all code and scripts used in this study.

#### Corpus

The complete corpus comprises close to 11 h of audio (and video) recordings from 46 participants (~ 30 min. per dyad). We focus in this analysis on the spoken modality ; analysis of gaze and other multimodal signals is in progress (see Section "Discussion").

#### Annotation, processing and measurement

For the annotation, processing and general analysis of data, we directly followed the most closely related previous study^8^ (which itself builds on established empirical work on turn-timing). Accordingly, not all details for all steps are explicitly laid out here—the relevant previous work and the openly shared scripts and code accompanying the current study exhaustively document all processing and analysis steps and decisions.

The speech data were annotated in *Praat*^29^ (version 6.4.20) by trained phoneticians into silent and non-silent intervals, with a minimum silence length of 200 ms. The corpus contains 13,346 transitions between speakers (after exclusion of 4 transitions for which the end of the speech turn had mistakenly not been labelled). For the analysis of turn-timing, within-speaker overlaps—where one speaker’s turn briefly overlaps with speech of their interlocutor without the floor subsequently being transferred to the other speaker—were excluded, following standard practice^1,8^. Within-overlaps do not entail a true floor transfer from one speaker to another and are therefore not of primary importance for an analysis of turn-transition timing. For completeness, we will give an overview of the comparative distribution of all transition types here. In the autistic group, there were proportionally 1) fewer within-speaker overlaps (18.1%) than in the non-autistic group (24.9%), 2) fewer between-speaker overlaps (19.6%) than in the non-autistic group (24.7%), and 3) more silent gaps (62.3%) than in the non-autistic group (50.4%).

**Fig. 1:**
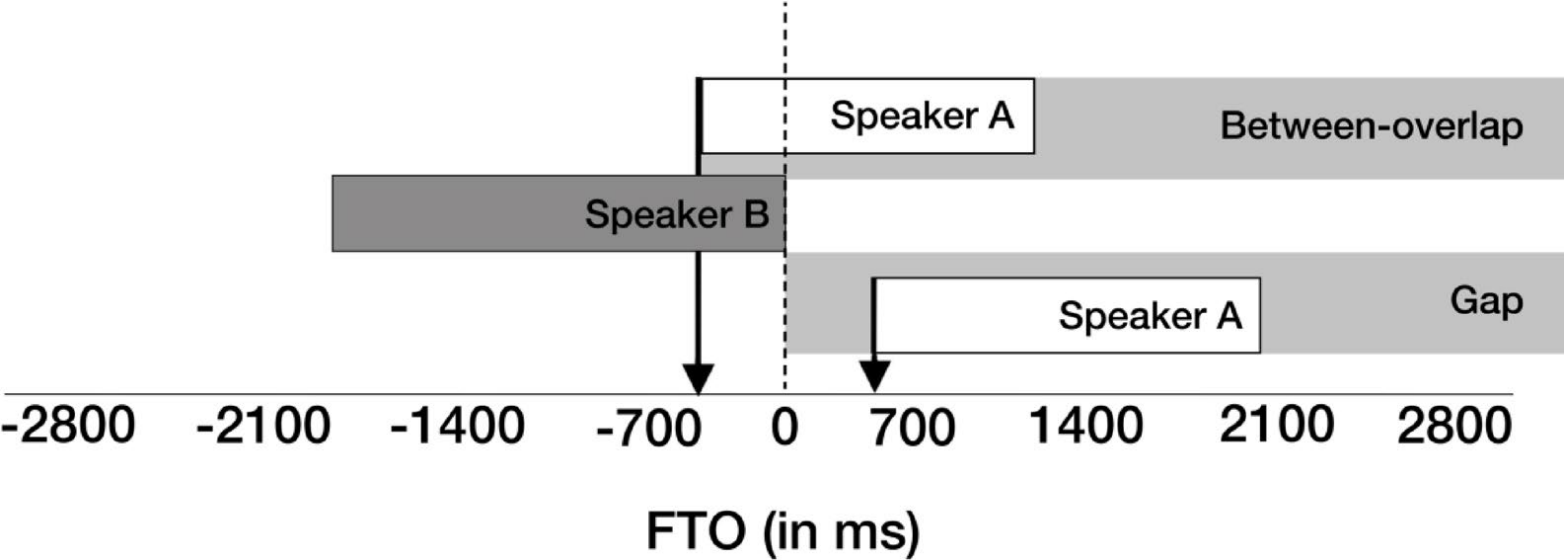
Categories and measurement of turn transitions. “Gaps” are silent intervals between turn transitions; “between-overlaps” are turn transitions composed of overlapping speech from both interlocutors. Gaps are represented with positive Floor Transfer Offset (FTO) values (see right arrow for an FTO value of about + 600 ms); overlaps are represented with negative FTO values (see left arrow for an FTO value of about − 600 ms). Reproduced from original article^8^ under a Creative Commons Attribution license (CC-BY 4.0).

The exclusion of within-speaker overlaps leaves 10,251 turn transitions for the main analysis. Of these transitions, 69% were gaps (silence during speaker transition) and 31% were overlaps (both speakers talking simultaneously during transition) across groups. Turn transitions were analysed using the measure of Floor Transfer Offset (FTO), in which positive values represent gaps and negative values represent overlaps between speakers (see Fig. 1). As FTO reflects the temporal coordination between interlocutors, it is analysed at the level of dyads rather than individuals.

#### Statistical analysis

We used Bayesian inferential modelling for statistical analysis. We consider effects as robust, or as providing compelling evidence, for which the 95% credible interval (CI) under the posterior distribution does not include zero, or only by a small margin, and for which the posterior probability (that a difference δ is greater than zero) is ≥ 0.89^30^ (but please note that any such cut-off is arbitrary^30^, and that incidentally the vast majority of robust effects reported in this work have a posterior probability of 1). We used regularising weakly informative priors for all models^31,32^. All priors were centred at zero and distributions were chosen according to relevant results in the literature (e.g. the feasible range of FTO values). Full posteriors are provided in the Supplementary Information.

We used Bayesian multilevel linear models implemented in the modelling language *Stan* (version 2.32.7)^33^ via the package *brms* (version 2.22.0)^34^ for the statistical computing language *R* (version 4.5.0)^35^, which we used in the software *RStudio* (version 2025.05.1)^36^. All models were fitted using an exponentially modified Gaussian (ex-Gaussian) likelihood. We performed posterior predictive checks with the packages *brms* and *bayesplot* (version 1.12.0)^37^, enabling us to confirm that the combinations of priors and likelihood were suited to the data set. For all models, four sampling chains ran for 4000 iterations with a warm-up period of 2000 iterations. Besides the packages for Bayesian modelling, we made extensive use of the packages included in the *tidyverse* collection for performing data import, tidying, manipulation, visualisation and programming^38^. Further details on Bayesian modelling can be found in the Supplementary Information; for an exhaustive and reproducible account, see also the repository at https://osf.io/3xm2w, where all code, scripts and (derived) data are available in an open-access repository for inspection and independent corroboration.

All models used FTO as the dependent variable, the interaction Group (ASD/non-ASD)*Task (Introduction/Tangram/Discussion) as a fixed factor, and Dyad as a random factor (wit random intercept and a random slope for Task). Since each dyad has a unique ID and appears in only one group, Dyad is not nested within Group. By default, group differences are reported with the ASD group as the reference level; context differences are reported with the Introduction as the reference level.

Three separate models were run to test the effect of group on FTO, a main model for the full FTO range, i.e. including both gaps and overlaps (positive and negative FTO values, respectively), plus one each for only gaps (positive FTO values) and only overlaps (negative FTO values). The standard operationalisation of turn-timing as FTO values results in a leptokurtic distribution peaking near 0 ms and a long right tail, but also including many negative values. Although it is possible to capture such data appropriately with an ex-Gaussian model^14^, we decided to additionally report and model gaps and overlaps separately for conceptual reasons. While the FTO measure is reliable and very helpful for illustrative purposes, we argue that overlaps and gaps are not necessarily treated as equivalent events on a continuous scale in perception and cognition. For instance, an overlapping transition of 200 ms should not be expected to constitute the “mirror image” of a silent gap transition of 200 ms^39,40^. A helpful analogy might be drawn to the measure of voice onset time (VOT) in phonetics. VOT distinguishes voiced plosives such as < b > from unvoiced plosives such as < p > on a continuous (millisecond) scale. At the same time, however, voiced and unvoiced plosives can be subject to categorical perception and are treated accordingly in most language and writing systems and phonological theories^41^. It is plausible that positive and negative FTO values might similarly reflect qualitatively different phenomena in conversation, motivating the separate, exploratory examination of gaps and overlaps in addition to the overall FTO distribution. Nevertheless, the output of the full FTO model provides the most comprehensive group comparison, not least because it includes all data points rather than a subset.

## Results

We will first describe group differences (ASD vs. non-ASD) in turn-timing across conversational contexts, and then turn to the effect of conversational context within and across groups.

### Longer silent gaps in the autistic group across conversational contexts

**Fig. 2:**
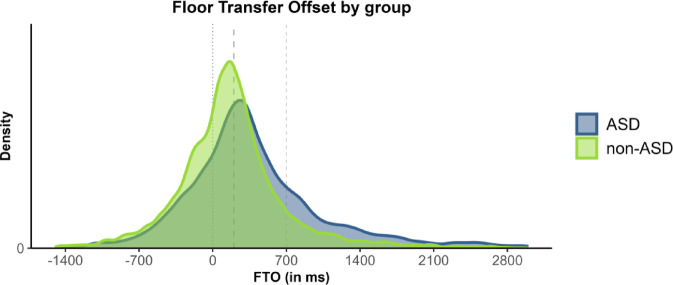
Density plot of Floor Transfer Offset (FTO) values by group (across conversational contexts). Positive values represent gaps; negative values represent overlaps. ASD group in blue, non-ASD group in green. The dotted line indicates the value of 0 ms FTO, representing no-gap-no-overlap transitions. Dashed lines indicate the values of 200 ms (expected for typical transitions) and 700 ms FTO (unusually long transitions).

We found a robust effect of consistently longer silent gaps in turn transitions between autistic speakers across conversational contexts (Fig. 2). The mean FTO value in the autistic group was 512 ms (SD = 1027), with a median of 307 ms. These values are considerably higher than typical values reported in the literature, and more than twice as high compared to the non-autistic group in this study, for which we found a mean of 188 ms (SD = 634) and a median of 149 ms.

**Fig. 3:**
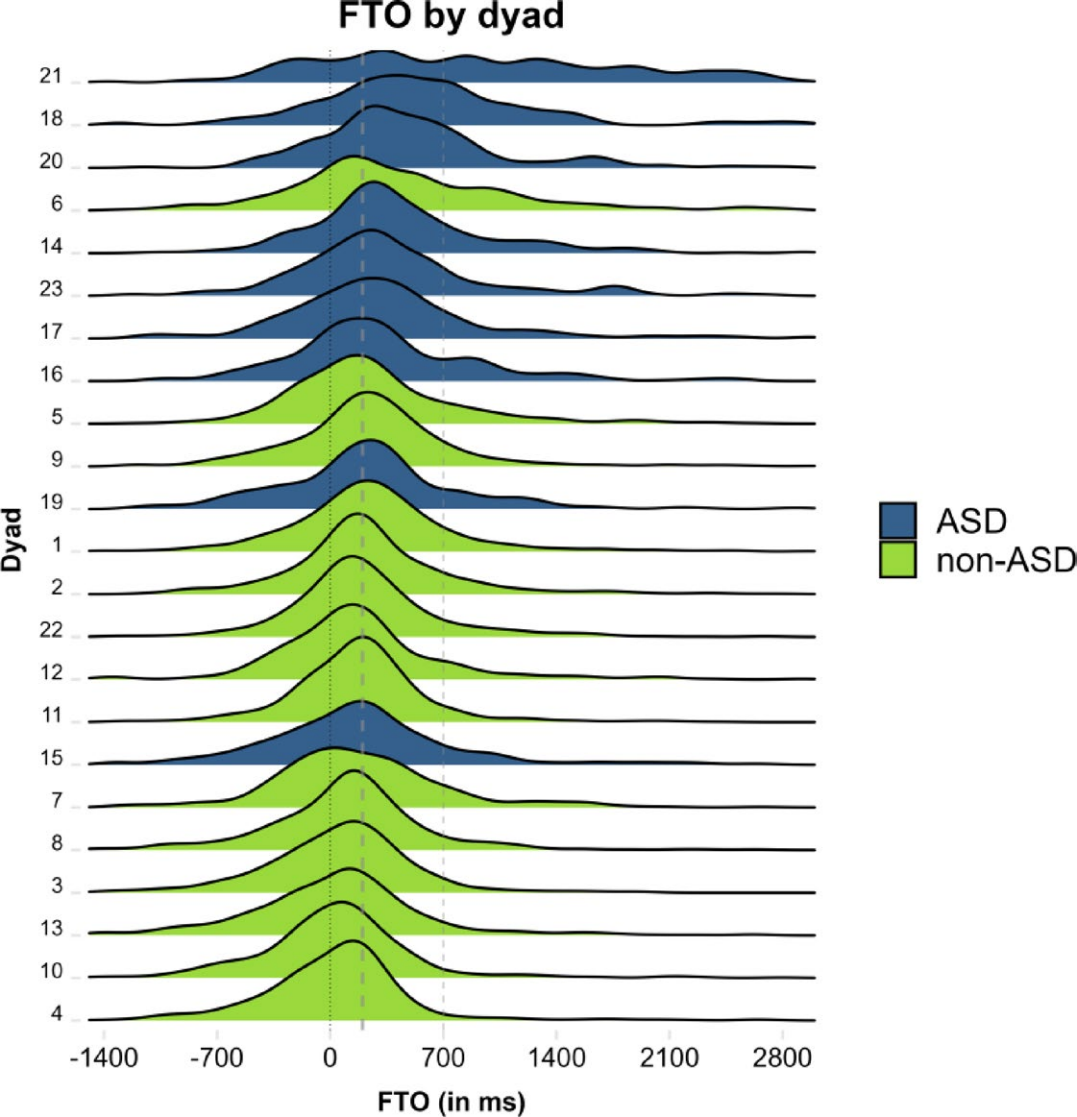
Density plot of Floor Transfer Offset (FTO) values by dyad and group (across conversational contexts). Positive values represent gaps; negative values represent overlaps. ASD group in blue, non-ASD group in green. The dotted line indicates the value of 0 ms FTO, representing no-gap-no-overlap transitions. Dashed lines indicate the values of 200 ms (expected for typical transitions) and 700 ms FTO (unusually long transitions).

Investigation at the dyad level revealed remarkably consistent behaviour, with very few speaker pairs deviating notably from the group-level pattern (compared to related previous analyses in which overlap between groups was substantial^7,21^). We can observe, for instance, that 7 out of the 8 highest mean FTO values were produced by autistic dyads, and, conversely, the 6 lowest mean FTO values were all produced by non-autistic dyads (Fig. 3). This does not negate the fact, however, that as in related analyses of dyad- and individual-specific behaviour, there was no clear and strict dividing line between groups. Indeed, certain dyads patterned more with dyads from a different group, as in the case of the non-autistic dyad with the highest mean FTO value (dyad 6) and the autistic dyad with the lowest mean FTO value (dyad 15); see Fig. 3.

Dyad 21 from the ASD group stands out not only by having the highest mean FTO value overall, but also with an unusually flat distribution of FTO values (see top of Fig. 3). We therefore decided to investigate whether general characteristics or other behavioural metrics might explain the unusual pattern in this dyad. Both speakers in Dyad 21 had similar values for verbal IQ (both 107), AQ (30; 27) and self-rated rapport (26; 23), all of which are close to the average values in the ASD group. The dyad completed a relatively low number of tasks (5) in the Tangram context, but had 100% accuracy (see section "Longer silent gaps and successful social adaptation in autism" for related discussion). Most importantly, however, Dyad 21 showed the lowest rate of backchannels (listener signals) per minute of any dyad in the corpus (1.6) and produced very little eye contact (close to zero)^42–44^. As both vocal feedback and mutual gaze have been proposed to play important roles in interaction and in turn-taking more specifically, we can speculate that the unusual metrics for turn-timing, feedback, and eye gaze in this dyad are correlated. We will test such speculations empirically in forthcoming work.

Finally, while turn-timing is specifically a dyad-level phenomenon, we can get a complementary sense of speaker-level contributions through an analysis of the relative speaking time within dyads (speaker balance)^18,45^ (calculated as % speaking time speaker 1 − % speaking time speaker 2). The lower the score, the more balanced the contributions from the two interlocutors. Briefly, speaker contributions were more balanced in the non-ASD group (mean = 9.83; SD = 9.4) than in the ASD group (mean = 12.9, SD = 8.75), in agreement with related findings^18^. Furthermore, 6 out of the 7 most balanced dyads were part of the non-ASD group. Interestingly, however, the least balanced dyad (Dyad 13), with a score of 35.7, was also non-autistic (see Figure S1 in the Supplementary Information). At the same time, the least balanced dyad Dyad 13 did not particularly stand out in turn-timing, with the third-lowest mean FTO value. Conversely Dyad 21, profiled above for having evinced very unusual turn-timing behaviour, did not stand out in terms of speaker balance, with a score of 16.1.

#### Statistical analysis

Bayesian inferential modelling confirms a robust effect of group (ASD vs. non-ASD) on turn-timing across conversational contexts.

The model output for FTO by group across conversational contexts reveals a mean difference (δ) of -204 (ms), with a 95% CI of [-339, -66] and a posterior probability of *P* (δ > 0) = 1. This represents evidence for a robust effect of group (diagnostic status) on FTO (turn-timing), with higher FTO values (longer silent gaps) for autistic dyads.

As laid out in Sect. 2.4, we complemented the main statistical model, which includes all FTO values (positive and negative), with two models which included either only gaps or only overlaps, respectively. In brief, the model of only gaps confirms a small but robust effect of group on turn-timing, as evidenced through a 95% CI excluding zero, and a posterior probability of *P* (δ > 0) = 1. The ex-Gaussian model of overlaps, on the other hand, does not provide evidence for a group effect, with a 95% CI including zero, and a posterior probability of *P* (δ > 0) = 0.81. The outcome of these models a) conclusively confirms the group difference in silent gap duration and b) suggests that overlap duration is not the decisive factor for the overall group difference in FTO values.

Finally, and leading over to the next section, we will summarise by-group comparisons for each separate conversational context. While, crucially, the group difference clearly proved robust for each context, with 95% CIs excluding zero and posterior probabilities of 0.99 or higher for all contexts, it is worth noting that, numerically, the difference between groups was greatest for the Introduction; see the following section for more detail.

### Longer silent gaps in the task-based compared to spontaneous contexts across groups

We found a robust effect of longer silent gaps in the Tangram compared to the two spontaneous conversational contexts (Introduction; Discussion) in the non-ASD group, and between the Tangram compared to the Discussion context (only) in the ASD group; see Table 2 for mean (SD) and median values. There is no clear or unidirectional difference between the Introduction and Discussion contexts overall based on summary statistics (see section "Statistical analysis" for results from inferential modelling). As noted above in the context of statistical modelling, summary statistics further suggest that the difference between groups was most pronounced for the Introduction, with a difference in mean FTO values of 355 ms. Perhaps a more expressive way of stating the same observation is that, for the Introduction, the mean FTO value of the ASD group was almost 5 times higher than that of the non-ASD group. For the Tangram and Discussion contexts, the group difference is expressed by a factor of less than 3. Notably, this entails that behaviour was robustly different between the spontaneous Introduction and the task-based Tangram context in the group of non-autistic dyads but not in the group of autistic dyads.

**Table 2:** Mean (SD) and median FTO values (in ms) by conversational context and group.

Group	Introduction			Tangram			Discussion		
	Mean	SD	Median	Mean	SD	Median	Mean	SD	Median
ASD	446	933	299	663	1188	370	403	887	265
Non-ASD	91	459	117	316	706	197	164	695	137

Although consistent, meaningful, and communicationally relevant, the differences in FTO values between the Tangram and the other contexts (within groups) are rather small numerically (range across groups: 155 to 228 ms). For this reason, we will in the following illustrate the effect of context through a categorical classification of transition types^18^, based on perceptual relevance and previous research. Any gaps or overlaps with an absolute duration of less than 100 ms were categorised as *smooth* transitions. Gaps or overlaps with an absolute duration of or exceeding 700 ms were categorised as *long gaps/overlaps*, and the remaining transitions with an absolute duration of 100–699 ms were categorised as short *gaps/overlaps*. The cut-off point at 700 ms was inferred from previous work showing that silences (though not necessarily between-speaker gaps) of 700 ms or longer are perceived as unusual by listeners and cue repair initiations or non-affiliating responses^46–49^.

**Fig. 4:**
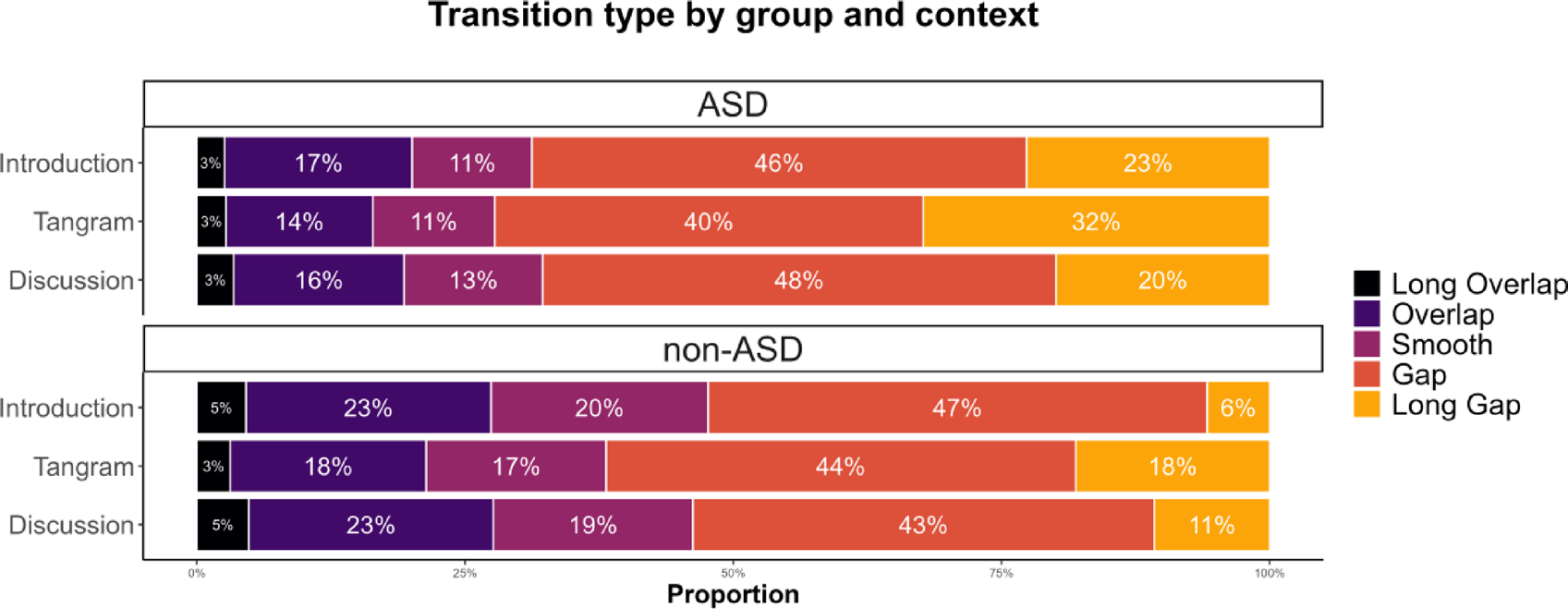
Stacked bar charts by group showing rounded proportions of different transition types. ASD group on top, non-ASD group below. Transition proportions on the x-axis: long overlap transitions (FTO ≤  − 700 ms) in black, overlaps (FTO -699 ms – -100 ms) in dark purple, very short (smooth) transitions (FTO -99 – 99 ms) in light purple, gaps (FTO 100 ms – 699 ms) in orange and long gaps (FTO ≥ 700 ms) in yellow.

As can be seen in Fig. 4, the Tangram context stands out in eliciting more long gaps (and fewer overlaps) in both groups. More generally, this representation acts as a visual corroboration of the analysis of group-level differences laid out above. It is immediately apparent that the ASD group produced more long gaps overall, and that the non-ASD group, conversely, produced more “smooth” transitions and overlaps than the ASD group.

Finally, we will point out that, in contrast to related earlier work^8^—where ASD dyads produced slower turn-timing than non-ASD dyads only in the first minutes of conversational interaction—we found no such effect of changes in turn-timing from the first stages to the remainder of interaction (see the accompanying repository for details of the analysis).

#### Statistical analysis

We used the same model as described in the beginning of Section "Statistical analysis" (encompassing all FTO values) to analyse effects of conversational context on FTO within groups. Output from the model provides evidence for a robust difference between the Tangram context compared to both the Introduction and Discussion contexts in the non-ASD group, with all 95% CIs excluding zero and all posterior probabilities at *P* (δ > 0) = 1, reflecting higher FTO values in the Tangram context. The only robust difference between contexts in the ASD group was found for the Tangram compared to the Discussion context, with a posterior probability of *P* (δ > 0) = 0.92 and a 95% CI only narrowly including zero. All further details can be found in the OSF repository at https://osf.io/3xm2w/.

## Discussion

### Summary

Our study provides the first evidence for consistently longer silent gaps in the conversations of adult autistic as compared to non-autistic speaker pairs. This group difference prevailed across the three different conversational contexts under investigation. Moreover, both groups produced slower turn-timing, in the form of longer silences, for the task-based (Tangram) compared to one or both free conversational contexts, broadly in line with related recent results^11^.

### Longer silent gaps and successful social adaptation in autism

Turn-timing was consistently slower in the group of inter-autistic dyads. At the same time, self-rated rapport, assessed via post-hoc questionnaires, shows that ASD dyads rated their subjective experience of the interaction only slightly lower than non-ASD dyads (ASD group mean = 25.19 (SD = 5.27); non-ASD group mean = 28.88 (SD = 3.64)). While this group difference is robust according to Bayesian modelling (mean δ = 3.78; 95% CI [1.1, 6.31]; *P* (δ > 0) = 0.99)^42^, it was our impression that the conversations between autistic interlocutors were enjoyable and rewarding, with some participants also showing high levels of reciprocal interest beyond the end of the experiment. In this light, it is important to note that standard questionnaire-based rapport ratings may not be an appropriate tool for capturing feelings of rapport as experienced by autistic people. Regarding task success in the Tangram context, accuracy (proportion of tangram sets correctly solved) was roughly equivalent between groups (ASD mean = 89.8%, SD = 11.1%; non-ASD mean = 92.5%, SD = 11.3%), but the non-autistic group completed slightly more Tangram sets overall in the allotted 10 min (mean = 10.8; SD = 4.85) than the autistic group (mean = 7.62; SD = 3.11). Bayesian binomial logistic regression models confirm a robust group effect for number of Tangram sets completed (mean δ = 0.62; 95% CI [0.29, 0.95]; *P* (δ > 0) = 1), but no robust group effect (despite a clear trend) for accuracy (mean δ = 0.81; 95% CI [-0.19, 1.85]; *P* (δ > 0) = 0.9). Clearly, however, success in the Tangram task is not an ideal measure of overall conversational success or efficiency. We are aiming to develop more appropriate and reliable measures of rapport and conversational success in future work, ideally targeted for and co-designed with autistic people.

In conjunction with related work, which has repeatedly shown that matched autistic dyads did not differ significantly from matched non-autistic dyads in terms of rapport, information transfer or related measures^15,17,50^, we interpret the pattern of slower turn-timing in autistic dyads as a successful, functional adaptation. Far from being inappropriate or even a “deficit”, the conversational rhythm found in the ASD group appears to be judged by these speaker pairs to be the rhythm that is most appropriate and most comfortable, an example of successful social calibration. While such an interpretation is speculative at this time, it leads to concrete predictions. For instance, autistic listeners might judge long silent gaps in conversations as less negative than non-autistic listeners. We are currently preparing a perception experiment^48^ with autistic listeners to test this prediction. More broadly and no less importantly, the lived experiences of autistic people have also indicated that they may be more comfortable with stretches of silence during conversational interaction^51^. This view of longer silences in inter-autistic dyads furthermore echoes previous evidence and accounts regarding a (seemingly deliberate) reduction in mutual gaze^42,43^ and verbal feedback^18,42,52^.

On the note of vocal feedback, we did not explicitly account for feedback signals like backchannels in the current analysis, but previous work tells us that around 70% of the within-overlaps excluded from the analysis can be expected to contain backchannels^8^. In a separate, in-depth analysis of feedback in the current data set (as yet unpublished), we found that autistic speakers produced far fewer vocal backchannels per minute (3.27) than non-autistic speakers (7.33)^42,44^, in line with previous results^52^. This supports the current analysis of turn-timing by suggesting that fully taking into account backchannels, which are less frequent in the ASD group and typically occur in overlap, would only strengthen the overall pattern of comparatively more silence and less overlapping talk in the ASD group that emerges from the current turn-timing results.

While most studies on inter-autistic interaction have found little or no differences in behaviour compared to non-autistic speaker pairs, the current study adds to previous evidence on backchannels^52^, filled pauses^53^ and intonation^54^ in finding clear group differences between matched dyads of autistic vs. non-autistic adults. We believe that a key factor is methodological in nature: all of the above studies reporting group differences analysed (semi-)spontaneous interaction, whereas the studies reporting no differences usually relied on data from highly structured, monologic speech (but see recent evidence for equivalent results across picture descriptions and small talk^55^).

This raises the question of how to interpret the group differences we observed in the current work. In the context of autism research, it is particularly important to not conflate behavioural differences with communicative difficulties^56^. Accordingly, we do not see the current results as standing in opposition to the highly valuable and influential notion of the Double Empathy Problem^57^ (DEP). The DEP emphasises that communication difficulties in autism tend to arise from misunderstandings in *mixed-*neurotype (non-ASD–ASD) interactions, and that such mismatches have been very frequently misinterpreted as autistic “deficits”. Importantly, we cannot expect difficulties arising in mixed interactions to extend to (the much less well-studied) *matched-*neurotype (ASD–ASD) interactions. Results showing differences in the behaviour of autistic as compared to non-autistic dyads are, however, fully compatible with this outlook. Indeed, we argue that identifying autism-specific patterns in communication can and should help us to promote mutual understanding and inclusivity, especially in cross-neurotype interaction, by making such preferences and strategies visible and interpretable, rather than pathologised.

This perspective is supported by the finding that the robust group-level differences we found nevertheless encompass considerable dyad-level variability, a fact highlighted both here and in previous related work^18^. We found, for instance, that turn-timing in one non-ASD dyad patterned with the ASD group, and vice versa. Individuals from both groups under study therefore clearly adjusted their behaviour according to the conversational context, their own preferences, and their interlocutor. This emphasises the crucial fact that conversational behaviour is shaped by specific interpersonal dynamics, which in turn strengthens situated, nuanced, and polythetic accounts of not only autistic communication but also human cognition more broadly^58^.

### Multimodal interaction, conversational context, and the universality of turn-timing

In addition to their relevance for autism research, our findings also speak to broader questions about conversational interaction. While the current results echo those of most previous work on turn-taking in autism, they stand in contrast to the most closely related previous study , which was the only one to also investigate matched dyads of autistic adults^8^. Although the same methods were employed in both studies, the previous work found no overall difference in turn-timing—only for the very first stages of the interaction. There might be several reasons for this discrepancy. Most importantly, the visual modality was blocked in the previous study, reducing the interaction to the spoken channel. In contrast, the conversational context, a collaborative (map) task in the previous work and the Tangram task here, was comparable in its structured nature (and recall that group differences persisted *across* contexts in the current analysis). The multimodal nature of interaction in the current setup could thus have been the decisive factor for the contrasting results, especially since we have found in a parallel analysis of the interactions under study here^42,43^ that eye contact (more specifically mutual gaze) was also reduced in the ASD group, as well as in the Tangram context for both groups.

Converging evidence for this interpretation centered on the role of visual signals comes from research into the perception and processing of multimodal cues in autism. Recent work shows that visual and auditory information seems to be processed differently in at least some autistic compared to non-autistic individuals^56^, specifically in the form of reduced preferential unconscious processing of eye gaze^59^ and possibly distinct neural mechanisms in autistic individuals^60^. While many of the pertinent claims are expressed within a deficit-focused paradigm, recent work importantly points out that autistic participants achieved similar outcomes while relying on different brain processes, and that “differences in brain processing in autism do not necessarily mean difficulties”^56^. A phenomenological perspective can fruitfully complement these experimental studies. This strand of research aligns with information from our own post-experiment interviews in showing a clear tendency for autistic people to subjectively experience eye gaze as often distressing or uncomfortable^42^. This perception can lead to the avoidance of direct eye gaze. In cross-neurotype interactions in particular, it may also prompt deliberate and effortful camouflaging strategies^61^.

Effects of conversational context are also important for the comparison of autistic and non-autistic speakers. The main finding of interest here is that the group-level difference was most pronounced in the Introduction context, where interlocutors engaged in spontaneous, unstructured conversation in order to get to know each other. This aligns with the often assumed and described tendency in autism for a discomfort with or dislike of typical (non-autistic) small-talk situations, although the literature on the topic is neither extensive nor conclusive^55,62,63^. A careful analysis of temporal dynamics *within* conversational contexts did not reveal any changes between the first moments of interaction and the remainder—contrary to previous results^8^. In this context it is important to acknowledge a confound: the first stages of interaction always took place within the Introduction context, and interactions even before the start of recording (and therefore analysis) were possible, even though such interactions were rare and, when they did occur, brief.

The current findings have important implications beyond the context of autism. First, we presented robust evidence for the observation that turn-timing was slower in the task-based Tangram compared to one or both spontaneous conversational contexts across groups, echoing recent results on child–adult interactions^11^. It seems reasonable to assume that the Tangram context was overall more cognitively demanding in requiring clear and explicit information transfer to achieve a set goal, since players were instructed to collaboratively solve a novel task to gain as many points as possible. This in itself may have led to a slower turn-timing rhythm with more and longer silent gaps. The Introduction context, on the other hand, was notable for featuring many overlapping turn transitions, potentially reflecting the more open-ended and affiliative character of these interactions.

While this observation of a context effect on turn-timing does not invalidate otherwise well-founded conceptions about potentially universal patterns of turn-timing across languages and cultures, it may serve as a note of caution. Certainly, we should remain open to the possibility that further research on different combinations of cultural, linguistic and social factors might qualify or narrow the scope of general or even universal turn-timing preferences^6,64^. Relatedly, the present results suggest that autistic dyads prefer a turn-timing pattern that differs from what has been described for other speakers of West Germanic languages, aligning instead with the upper range of FTO values found in cross-linguistic comparison^2^.

This consideration recalls a previous point: while turn-timing was slower in autistic dyads, this appears to have been an appropriate and functional choice, resulting in comfortable and successful communicative interaction. This warrants further investigation into rapport and communicative success, including an analysis of interactional repair. It is intriguing to speculate that (other-)repair may have been less frequent in the ASD group, based on suggestions that turn-taking and repair are interdependent, as well as previous analyses showing that autistic speakers tended to adhere more closely to assigned conversational roles and exchanged fewer but longer speaking turns^18,65,66^. Further avenues for future research are discussed in the following section.

### Limitations and horizons

As with any study, there are important limitations to the current work, and consequently its interpretation. We will list the most important limitations here, focusing on how they may inform future research with the potential to deepen, clarify or challenge the findings put forward in the present paper.

First, while we recorded a corpus of multimodal interaction, including the use of dual mobile eye-tracking glasses, an in-depth discussion of results from the visual domain is beyond the scope of this work. We made sure to summarise the most relevant findings on eye gaze where this enhanced the understanding and interpretation of the present analysis of turn-timing in spoken interaction. We are aware of the important role of visual signals in face-to-face interaction, and dedicated accounts of eye gaze and on the interplay of visual and spoken signals in the current corpus are in preparation, with further analyses of hand and head gestures also currently in progress. In the spoken domain, we are planning to also analyse the roles played by intonation and speech rate for turn-taking. Additionally, the decision to employ three conversational contexts in a fixed order, while indispensable for cross-speaker comparison and our experimental design (e.g. the Introduction has to occur when people meet for the first time; the Discussion of the Tangram task has to take place after completion of the task), entails that we cannot fully disentangle effects arising from the time course of the interaction in itself from specific effects of conversational context. Also note that while we report a basic analysis of performance in the Tangram task, we did not expressly assess participants’ general problem-solving or visuo-spatial skills, which are likely to be an important factor in this regard. Additionally, we are currently performing analyses of self- as well as other-rated rapport in the interactional corpus under investigation, which will result in a clearer picture of the correlation between conversational metrics and perceived rapport across and within groups.

Second, we are preparing to record mixed dyads, consisting of one autistic and one non-autistic interlocutor, with the same experimental setup as used for the recording of matched dyads investigated here. This will enable a dedicated comparison of mixed vs. matched dyads, in line with important recent work on the topic in other corpora (and languages). Relatedly, it is important to note that the sample of autistic individuals we investigated in this work is not representative of the autism spectrum as whole, with the various requirements inherent in the experimental setup (e.g. participation in an extended study of face-to-face interaction) as well as our inclusion criteria (e.g. diagnosis in adulthood at the University Hospital) acting as a narrow-band filter which leaves us with data from one particular region of the spectrum.

Third, we are planning to apply our analysis pipeline to comparable data from other European languages involving autistic individuals, to enable cross-linguistic and cross-cultural comparison, an aspect that is still severely understudied in the context of autism. It is of general importance to attempt to replicate previous studies and analyses, particularly when working with a limited population such as individuals on the autism spectrum^17^. For instance, while in section "Multimodal interaction, conversational context, and the universality of turn-timing" we gave our best explanation for the diverging results between the current and the most closely related previous study on turn-timing in autistic adults, it is entirely possible that this difference between studies is at least partly due to speaker- and dyad-specific behaviour. The current study analysed 18 autistic speakers, the previous study 14. While this is a high number compared to most quantitative research on conversational behaviour in autistic adults (simply due to the ~ 2% overall prevalence of autism and ensuing challenges in recruitment), it is of course still a very limited sample size in general terms. While Bayesian modelling provides evidence for a robust group difference in the current corpus, replication in future studies would therefore doubtless strengthen the present findings.

## Conclusion

The present results show that turn-taking in face-to-face interaction was consistently characterised by longer silent gaps in autistic dyads. Additionally, silent gaps were longer in the task-based compared to free conversational contexts, in both groups. These findings demonstrate that the temporal organisation of conversation varies according to interpersonal and situational factors, challenging the notion that there is one fixed target for turn-timing. Instead, speakers seem to adapt their conversational rhythm according to the communication style of their interlocutor and to contextual demands. Crucially, the difference in turn-timing observed for the group of autistic dyads may reflect successful social calibration, with all speaker pairs adjusting silent-gap length to strike a balance between interactional preferences and communicative efficiency.

## Supplementary Information

Supplementary Information.

## Data Availability

All data and scripts are available in OSF repositories. The relevant pre-registration can be found at https://osf.io/eum4n. The experimental data as well as scripts used to produce all plots, analyses and modelling reproduced and referred to in this article can be found at https://osf.io/3xm2w/. Metadata as well as further details on the experimental paradigm and related multi-modal measures can be found at https://osf.io/z5crx/.
