# Supplementary material for: Consistently longer silent gaps in autistic speaker pairs across three conversational contexts
Source: Sci Rep. 2026 May 15;16:15218. doi: 10.1038/s41598-026-53132-z (PMC13179315; doi:10.1038/s41598-026-53132-z)
Supplement: Supplementary file 1 — Supplementary Information. [file 41598_2026_53132_MOESM1_ESM.pdf]

Supplementary Information

Wehrle, Spaniol, Vogeley & Grice: "Consistently longer silent gaps in autistic speaker pairs across three conversational contexts"

1) Speaker balance

Figure S1. Balance of speaking time within dyads. The lower the score, the more balanced the speaking time. ASD group in blue, non-ASD group in green.

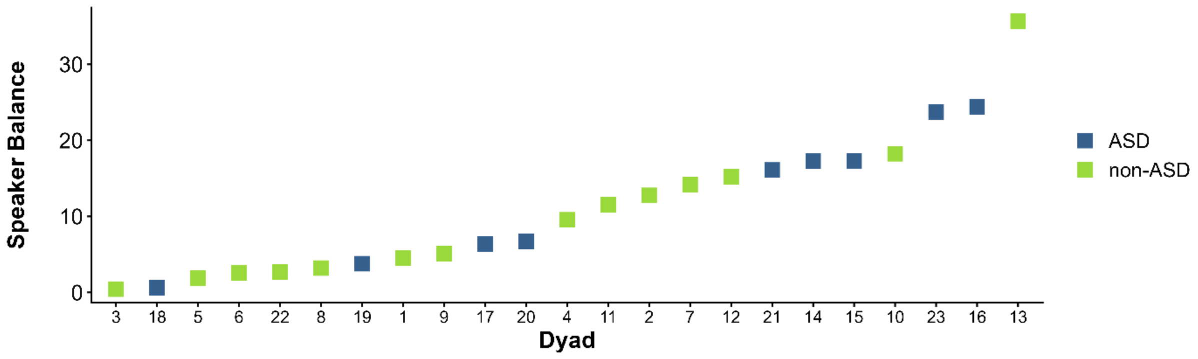

2) Bayesian model diagnostics and full posteriors plots

2.1) Full model (all FTO values)

2.1.1) Bayesian model diagnostics

Model specification:
Dependent variable: Floor Transfer Offset (FTO; full range, shifted to non-negative values)
Fixed effects: Group (ASD / non-ASD) × Task (Introduction / Tangram / Discussion)
Random effects: Dyad (random intercept)
Model family / likelihood: ex-Gaussian

Note that we converted milliseconds to seconds for modelling as it provided better performance, later transforming back to milliseconds for the write-up of the model output for better interpretability.

Prior distributions: Weakly regularizing, centered at 0

- Regression coefficients (b): Normal(0, 1)
- Intercept: Normal(0, 6)
- Sigma: Exponential(1)
- Beta (tail parameter): Exponential(1)

MCMC sampling details:
Sampler: No-U-Turn Hamiltonian Monte Carlo (NUTS)
Chains: 4
Iterations per chain: 4000
Warm-up per chain: 2000
Total post-warmup draws: 8000

No divergent transitions or other warnings observed.

Convergence and effective sample size:
Maximum R-hat across all parameters: 1.00
Minimum effective sample size: 1244
Mean effective sample size: 3000 (approx.)

Bulk and tail ESS values for individual parameters ranged from ~1244 to ~5332, indicating sufficient sampling efficiency and precision.

2.1.2) Full posterior plots

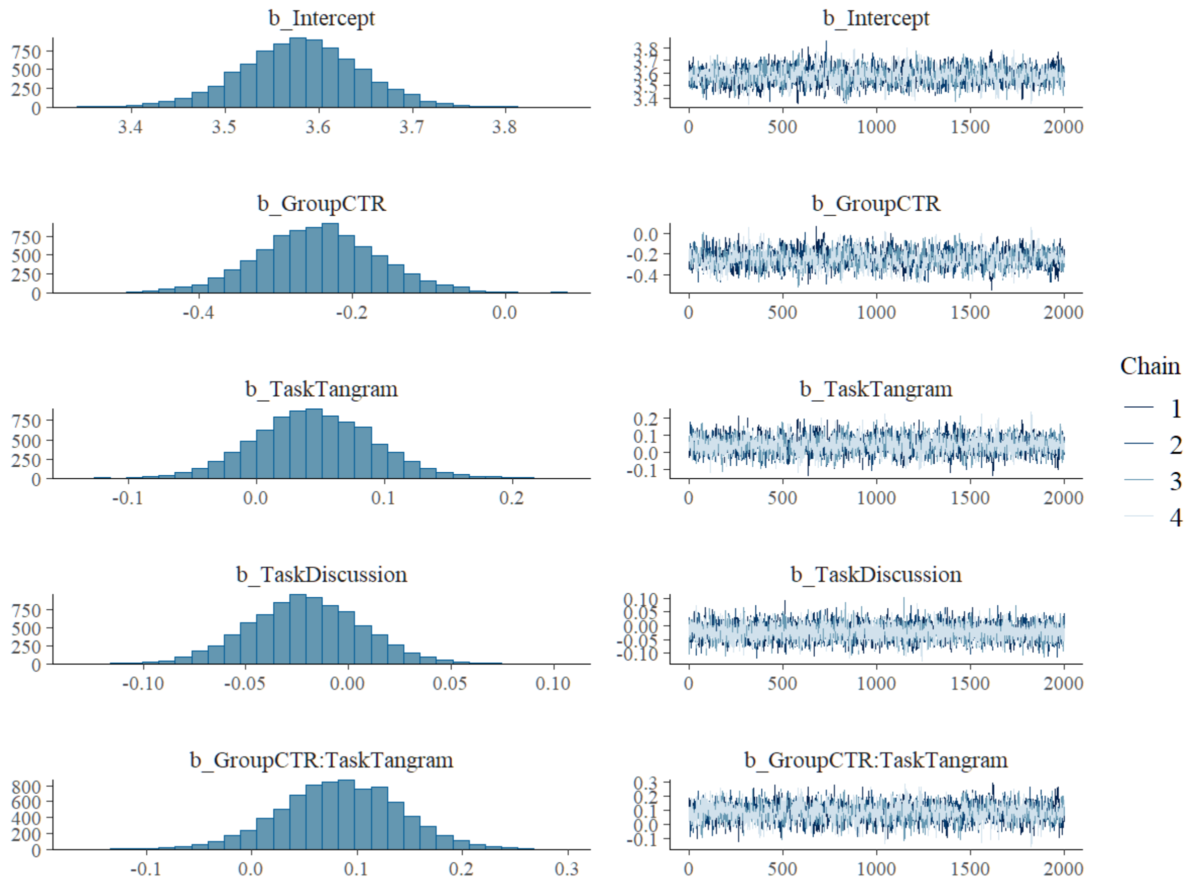

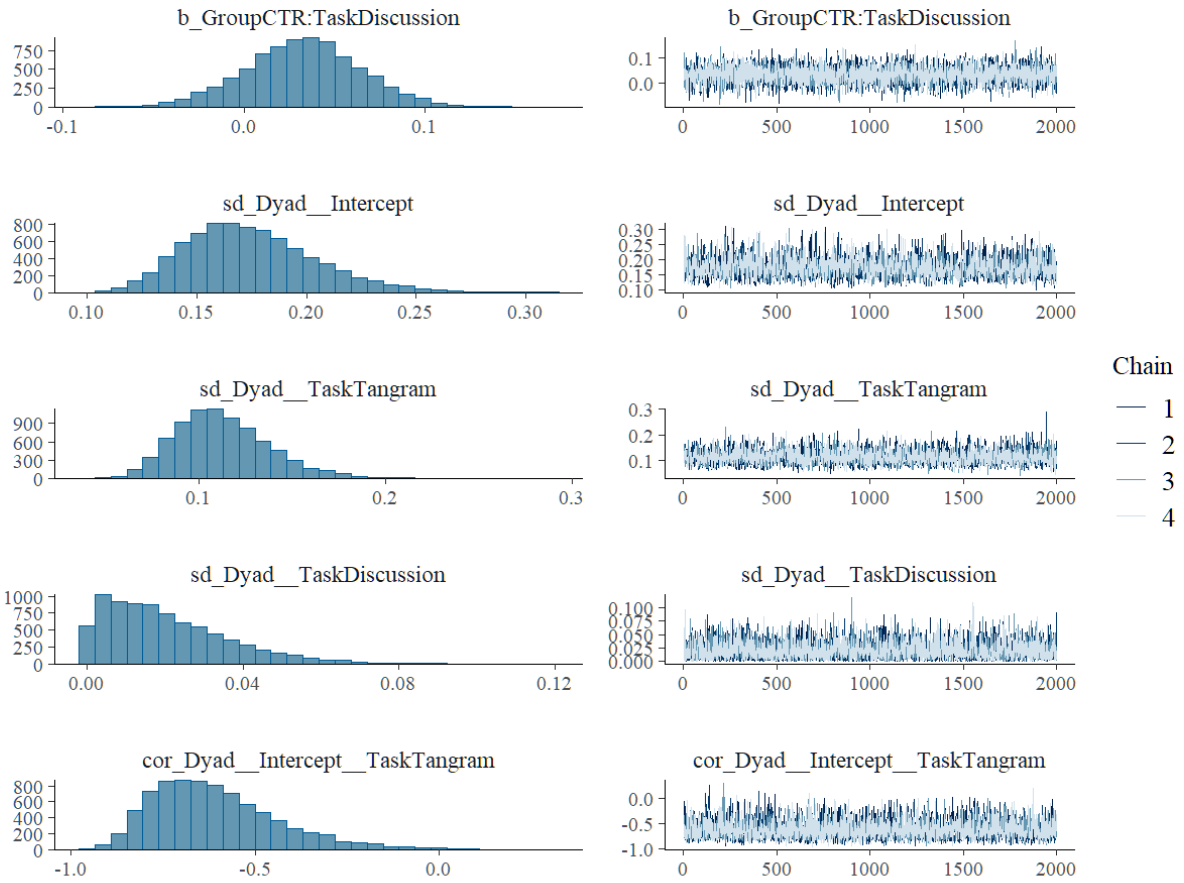
b_GroupCTR:TaskDiscussion
b_GroupCTR:TaskDiscussion
sd_Dyad__Intercept
sd_Dyad__Intercept
sd_Dyad__TaskTangram
sd_Dyad__TaskTangram
sd_Dyad__TaskDiscussion
sd_Dyad__TaskDiscussion
cor_Dyad__Intercept__TaskTangram
cor_Dyad__Intercept__TaskTangram
Chain
1
2
3
4

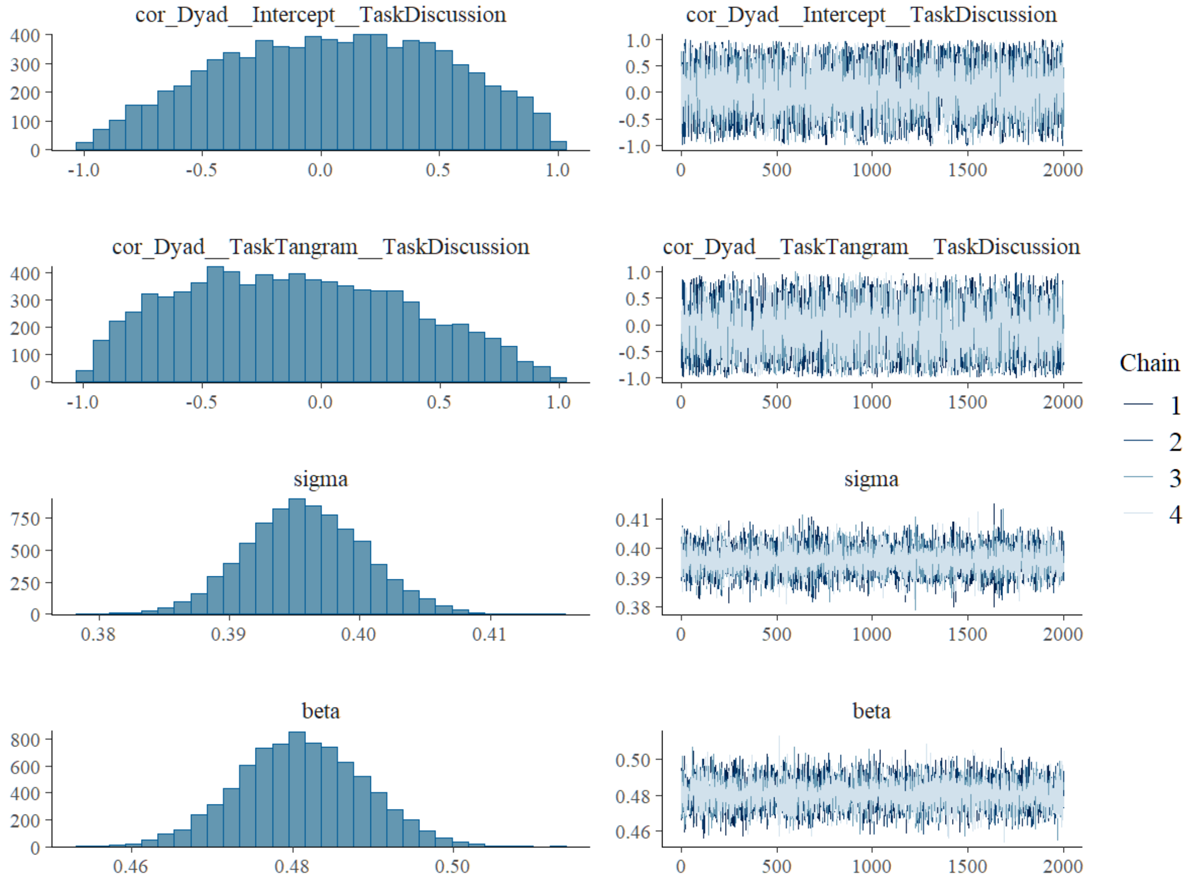
cor_Dyad__Intercept__TaskDiscussion
cor_Dyad__Intercept__TaskDiscussion
cor_Dyad__TaskTangram__TaskDiscussion
cor_Dyad__TaskTangram__TaskDiscussion
sigma
sigma
beta
beta
Chain
1
2
3
4

2.2) Gaps-only model

2.2.1) Bayesian model diagnostics

Model specification:
Dependent variable: Floor Transfer Offset (FTO; gaps only)
Fixed effects: Group (ASD / non-ASD) × Task (Introduction / Tangram / Discussion)
Random effects: Dyad (random intercept and slope for Task)
Model family / likelihood: ex-Gaussian

Note that we converted milliseconds to seconds for modelling as it provided better performance, later transforming back to milliseconds for the write-up of the model output for better interpretability

Prior distributions: Weakly regularizing, centered at 0

- Regression coefficients (b): Normal(0, 1)
- Intercept: Normal(0, 6)
- Sigma: Exponential(1)
- Beta (tail parameter): Exponential(1)

MCMC sampling details:
Sampler: No-U-Turn Hamiltonian Monte Carlo (NUTS)
Chains: 4
Iterations per chain: 4000
Warm-up per chain: 2000
Total post-warmup draws: 8000

A very small number (5) of divergent transitions (<0.1% of total draws) were observed; diagnostic checks confirmed these did not materially affect posterior estimates.

Convergence and effective sample size:
Maximum R-hat across all parameters: 1.00
Minimum effective sample size: 950
Mean effective sample size: 3000 (approx.)

Bulk and tail ESS values for individual parameters ranged from ~950 to ~5892, indicating sufficient sampling efficiency and precision.

2.2.2) Full posterior plots

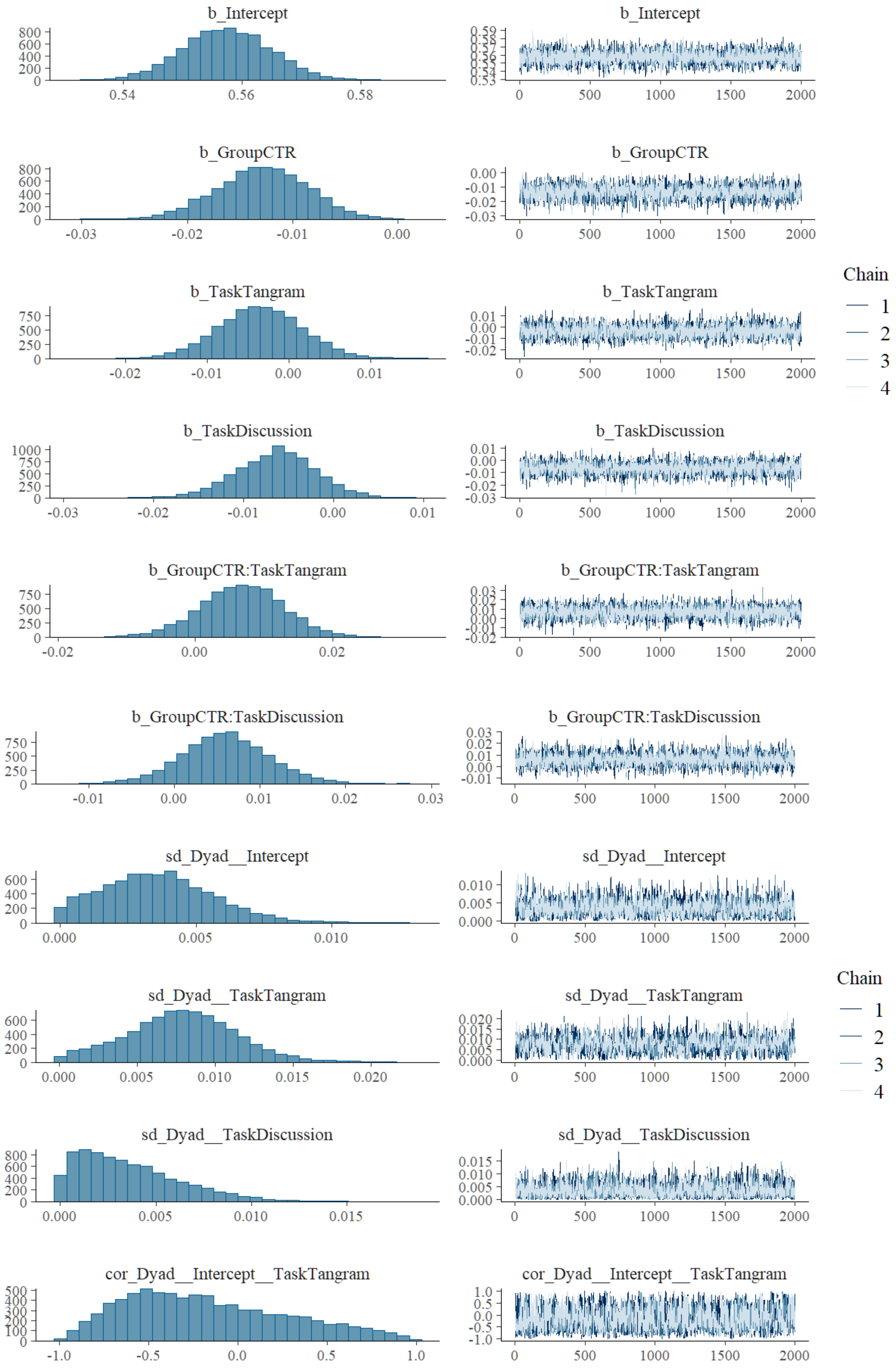
b_Intercept
b_Intercept
b_GroupCTR
b_GroupCTR
b_TaskTangram
b_TaskTangram
b_TaskDiscussion
b_TaskDiscussion
b_GroupCTR:TaskTangram
b_GroupCTR:TaskTangram
b_GroupCTR:TaskDiscussion
b_GroupCTR:TaskDiscussion
sd_Dyad__Intercept
sd_Dyad__Intercept
sd_Dyad__TaskTangram
sd_Dyad__TaskTangram
sd_Dyad__TaskDiscussion
sd_Dyad__TaskDiscussion
cor_Dyad__Intercept__TaskTangram
cor_Dyad__Intercept__TaskTangram
Chain
1
2
3
4
Chain
1
2
3
4

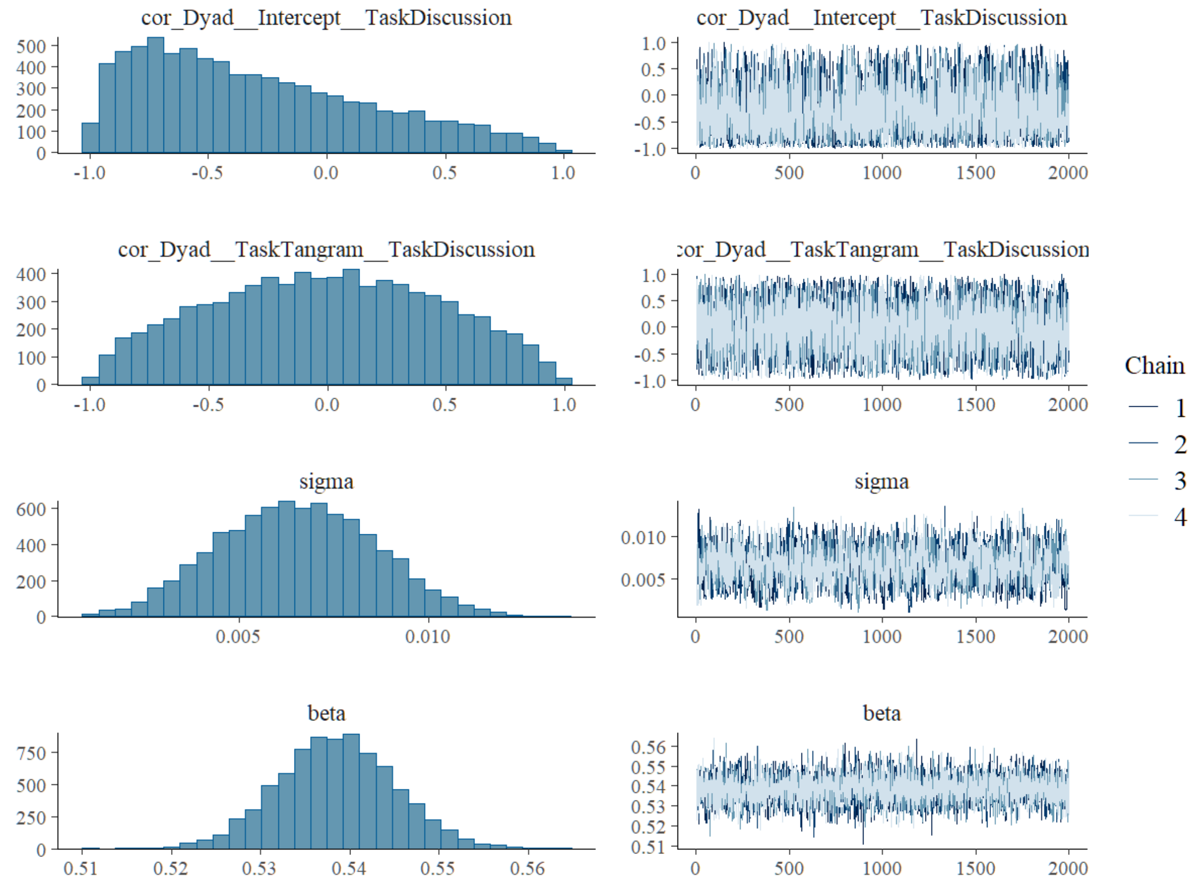

2.3) Overlaps-only model

2.2.3) Bayesian model diagnostics

Model specification:
Dependent variable: Floor Transfer Offset (FTO; overlaps only, absolute values)
Fixed effects: Group (ASD / non-ASD) × Task (Introduction / Tangram / Discussion)
Random effects: Dyad (random intercept and slope for Task)
Model family / likelihood: ex-Gaussian

Note that we converted milliseconds to seconds for modelling as it provided better performance, later transforming back to milliseconds for the write-up of the model output for better interpretability

Prior distributions: Weakly regularizing, centered at 0

- Regression coefficients (b): Normal(0, 0.5)
- Intercept: Normal(0, 3)
- Sigma: Exponential(1)
- Beta (tail parameter): Exponential(1)

MCMC sampling details:
Sampler: No-U-Turn Hamiltonian Monte Carlo (NUTS)
Chains: 4
Iterations per chain: 4000
Warm-up per chain: 2000
Total post-warmup draws: 8000

A very small number (29) of divergent transitions (<0.5% of total draws) were observed; diagnostic checks confirmed these did not materially affect posterior estimates

---

Convergence and effective sample size:
Maximum R-hat across all parameters: 1.00
Minimum effective sample size: 1863
Mean effective sample size: 4000 (approx.)

Bulk and tail ESS values for individual parameters ranged from ~1863 to ~5586, indicating sufficient sampling efficiency and precision.

2.3.2) Full posterior plots

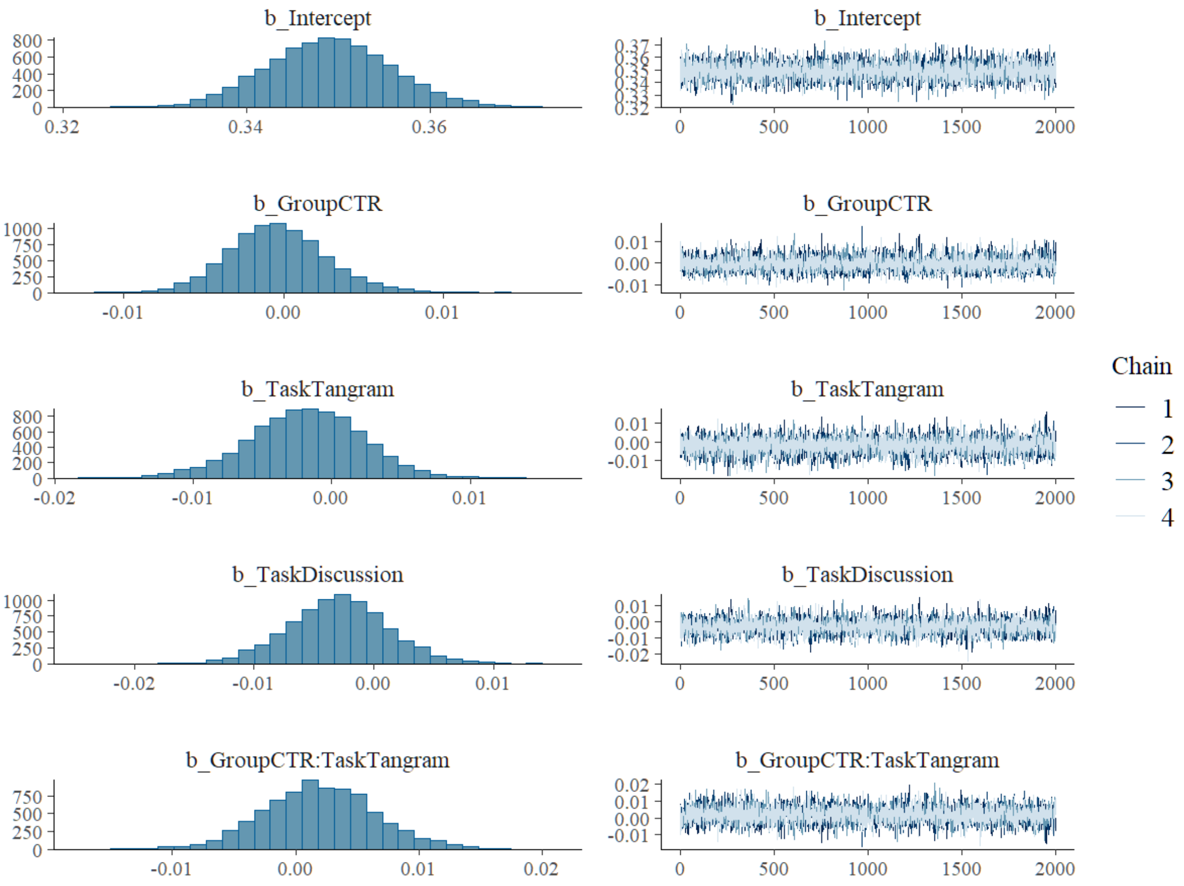

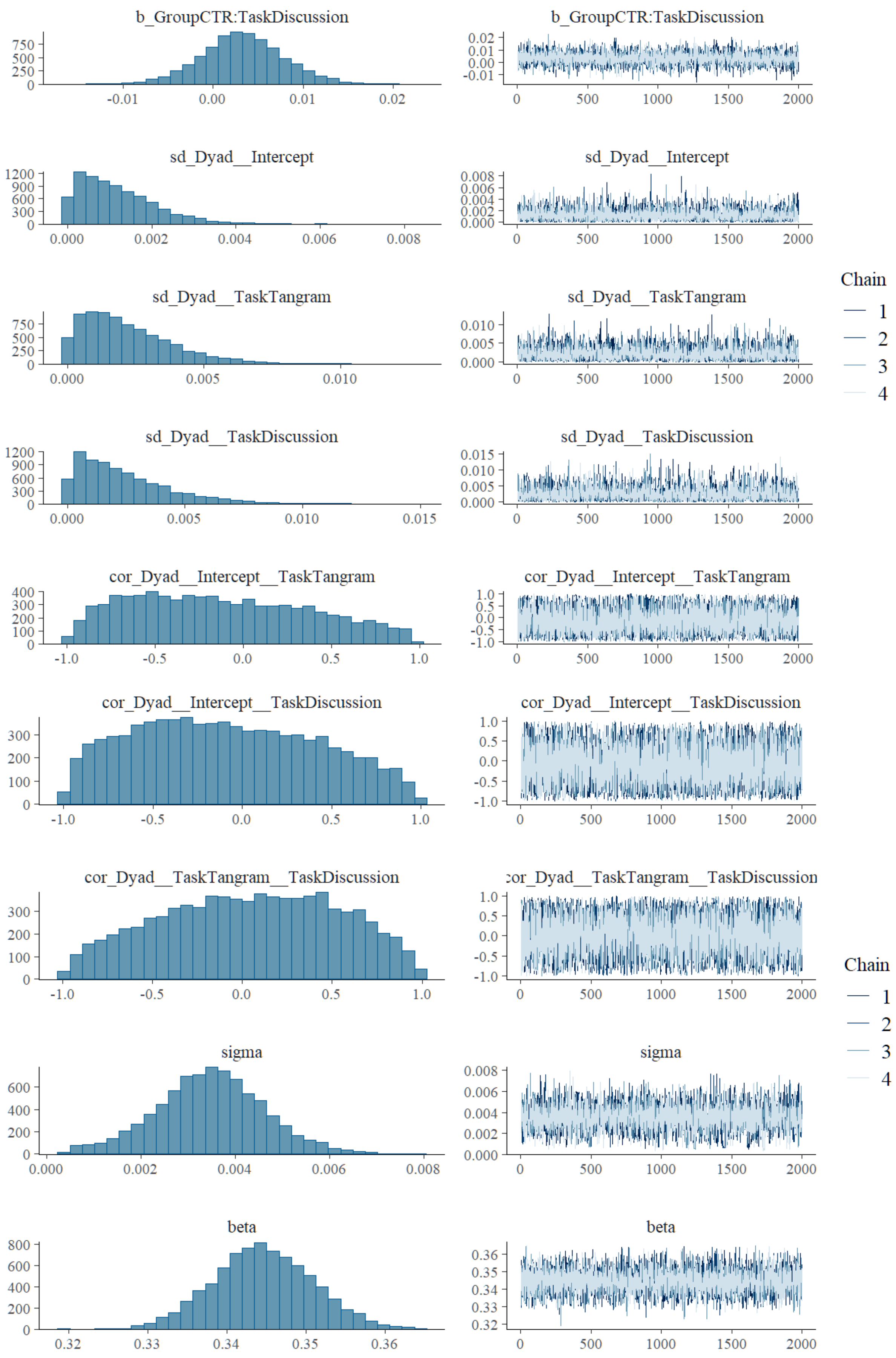
b_GroupCTR:TaskDiscussion
b_GroupCTR:TaskDiscussion
sd_Dyad__Intercept
sd_Dyad__Intercept
sd_Dyad__TaskTangram
sd_Dyad__TaskTangram
sd_Dyad__TaskDiscussion
sd_Dyad__TaskDiscussion
Chain
1
2
3
4
cor_Dyad__Intercept__TaskTangram
cor_Dyad__Intercept__TaskTangram
cor_Dyad__Intercept__TaskDiscussion
cor_Dyad__Intercept__TaskDiscussion
cor_Dyad__TaskTangram__TaskDiscussion
cor_Dyad__TaskTangram__TaskDiscussion
sigma
sigma
beta
beta
Chain
1
2
3
4
